# Impact of Diabetes and Metformin Use on Enteropancreatic Neuroendocrine Tumors: Post Hoc Analysis of the CLARINET Study

**DOI:** 10.3390/cancers14010069

**Published:** 2021-12-23

**Authors:** Sara Pusceddu, Claudio Vernieri, Massimo Di Maio, Natalie Prinzi, Martina Torchio, Francesca Corti, Jorgelina Coppa, Roberto Buzzoni, Maria Di Bartolomeo, Massimo Milione, Benjamin Regnault, Xuan-Mai Truong Thanh, Vincenzo Mazzaferro, Filippo de Braud

**Affiliations:** 1 Department of Medical Oncology, Fondazione IRCCS Istituto Nazionale Tumori (ENETS Center of Excellence), 20133 Milan, Italy; claudio.vernieri@istitutotumori.mi.it (C.V.); natalie.prinzi@istitutotumori.mi.it (N.P.); Martina.Torchio@istitutotumori.mi.it (M.T.); francesca.corti@istitutotumori.mi.it (F.C.); maria.dibartolomeo@istitutotumori.mi.it (M.D.B.); massimo.milione@istitutotumori.mi.it (M.M.); filippo.debraud@istitutotumori.mi.it (F.d.B.); 2 IFOM, The FIRC Institute of Molecular Oncology, 20139 Milan, Italy; 3 Department of Oncology, University of Turin, A.O. Ordine Mauriziano, 10124 Turin, Italy; massimo.dimaio@unito.it; 4 Department of Gastro-Entero-Pancreatic Surgical and Liver Transplantation, Fondazione IRCCS Istituto Nazionale Tumori (ENETS Center of Excellence), 20133 Milan, Italy; Jorgelina.Coppa@istitutotumori.mi.it; 5 Oncology, Clinica San Carlo Paderno Dugnan, 20037 Milan, Italy; roberto.buzzoni@clinicasancarlo.it; 6 First Division of Pathology, Department of Pathology and Laboratory Medicine, IRCCS Foundation National Cancer Institute, 20133 Milan, Italy; 7 Ipsen, 91940 Les Ulis, Ile-de-France, France; benjamin.regnault.ext@ipsen.com; 8 Ipsen, 92100 Boulogne-Billancourt, Ile-de-France, France; xuan-mai.truong@ipsen.com; 9 Department of Surgery, Fondazione IRCCS Istituto Nazionale Tumori (ENETS Center of Excellence), 20133 Milan, Italy; Vincenzo.Mazzaferro@istitutotumori.mi.it; 10 Oncology, Università Degli Studi di Milano, 20122 Milan, Italy

**Keywords:** diabetes mellitus, progression-free survival, lanreotide

## Abstract

**Simple Summary:**

Lanreotide was found to be effective for preventing tumors from worsening in patients with neuroendocrine tumors of the intestines and pancreas, regardless of whether or not patients also had diabetes. Metformin, a drug for treating diabetes, also seemed to prevent tumors from worsening.

**Abstract:**

The prognostic role of diabetes mellitus (DM) in advanced enteropancreatic neuroendocrine tumors (NETs) is unclear. Progression free survival (PFS) was assessed in post-hoc analyses of the 96-week, phase III, double-blind, placebo-controlled CLARINET study of lanreotide 120 mg in patients with advanced non-functional enteropancreatic NETs with DM (with/without metformin) and without DM. Of 204 patients, there were 79 with DM (lanreotide, *n =* 42 {metformin, *n =* 14}; placebo, *n =* 37 {metformin, *n =* 10}) and 125 without DM (lanreotide, *n =* 59; placebo, *n =* 66). Median PFS was 96.0 and 98.0 weeks with and without DM, respectively (hazard ratio 1.20 {95% confidence interval 0.79 to 1.82}; *p* = 0.380). No difference in PFS was observed in lanreotide-treated patients with/without DM (*p* = 0.8476). In the placebo group, median PFS was numerically shorter with versus without DM (*p* = 0.052) and was significantly longer in patients with DM and metformin (85.7 weeks) versus without metformin (38.7 weeks; *p* = 0.009). Multivariable Cox analyses showed that DM at baseline was not associated with PFS (*p* = 0.079); lanreotide was significantly associated with lower disease progression risk (*p* = 0.017). Lanreotide efficacy was confirmed in patients with advanced enteropancreatic NETs, regardless of diabetic status; DM was not a negative prognostic factor. A potential antitumor effect of metformin was observed in patients receiving placebo.

## 1. Introduction

Diabetes mellitus (DM) is an established risk factor for the development of pancreatic (pan) neuroendocrine tumors (NETs) [1,2,3]. In addition, obesity and metabolic syndrome have emerged as risk determinants for the development of well-differentiated gastroenteropancreatic NETs [4]. However, the prognostic impact of DM in patients with advanced enteropancreatic NETs is much less defined.

In the large, retrospective PRIME-NET study, patients with advanced panNETs with DM who were being treated with metformin were found to have significantly longer progression-free survival (PFS) when compared with both patients with DM not taking metformin, and those without DM [5]. Of note, the PRIME-NET study included patients treated with everolimus and/or somatostatin analogs, and the association between metformin use and improved PFS was independent of the concomitant antitumor therapy [5]. Therefore, DM might not affect the prognosis of patients with advanced panNETs, while the use of metformin could potentiate the activity of standard anti-panNET therapies [5].

Metformin is a widely prescribed antidiabetic drug with established efficacy and a favorable safety profile, and it is available at low cost [6]. Interestingly, several preclinical and retrospective studies revealed a potential antitumor effect of metformin, the potential impact of which in cancer prevention and/or treatment is now being investigated in prospective clinical studies [7]. Metformin exerts systemic metabolic effects, including the reduction of blood glucose and insulin concentration, which may result in indirect antiproliferative effects due to the modulation of insulin secretion and insulin-like growth factor-1 (IGF1) receptor (IGF1R) activity [8,9]. This, in turn, may lead to the downregulation of the phosphoinositide 3-kinase (PI3K)/protein kinase B (Akt)/mammalian target of rapamycin (mTOR) complex 1 (mTORC1) pathway in cancer cells [10]. Moreover, in preclinical studies, metformin has demonstrated cell-autonomous (direct) antitumor effects that are mediated by the inhibition of mitochondrial oxidative phosphorylation, which triggers the activation of adenosine monophosphate-activated kinase (AMPK) and suppresses anabolic processes and growth signaling mediated by mTORC1 [8,9]. Whether indirect or direct antitumor mechanisms are principally responsible for the in vivo antitumor effects of metformin remains to be elucidated [11]. The anticancer effects of metformin are a topic of interest across a number of cancer types, including colorectal, breast, prostate, and thyroid cancers [12,13,14,15,16,17]. Metformin-mediated inhibition of mTORC1 establishes a strong preclinical rationale for the synergistic or additive antitumor activity of metformin and mTORC1 inhibitors (such as everolimus), or somatostatin analogs, which also result in the inhibition of the PI3K/Akt/mTORC1 axis through the activation of somatostatin receptors [10].

Somatostatin analogs, such as lanreotide autogel/depot (lanreotide), are recommended as a first-line therapy in patients with well- or moderately-differentiated midgut NETs and panNETs [18]. In the phase III CLARINET trial, lanreotide, administered at a dose of 120 mg every four weeks, was found to reduce the risk of disease progression or death when compared with placebo in patients with advanced, non-functional enteropancreatic NETs [19]. Only a minority of patients had progressive disease (PD) at enrollment, while 96% of patients had stable disease at baseline radiological assessment, according to RECIST v1.0 [19]. Therefore, CLARINET enrolled a subset of patients with enteropancreatic NET who had particularly indolent disease.

In the present post hoc analysis of the CLARINET study, we investigated the impact of DM and metformin use on PFS in patients with advanced enteropancreatic NETs treated with placebo or lanreotide.

## 2. Materials and Methods

### 2.1. Overview of the CLARINET Study Design and Study Treatment

CLARINET was a phase III, international, randomized, double-blind, placebo-controlled, parallel-group, multicenter study (EudraCT: 2005-004904-35; ClinicalTrials.gov : NCT00353496 accessed on 10 November 2021). The full details of the study methodology and results have been published [19].

In brief, eligible participants were adults (aged ≥18 years) with advanced (American Joint Committee on Cancer stage IV), non-functional enteropancreatic, somatostatin receptor-positive NETs (originating in the pancreas, midgut or hindgut, or of unknown origin). Tumors were required to be well or moderately differentiated, as defined by a proliferation index (Ki67) <10% (or a mitotic index of ≤2 mitoses per 10 high-power fields, if the Ki67 could not be quantified reliably). Patients were excluded from the study if they had received prior treatment with a somatostatin analog (unless administered more than six months before treatment initiation and for a total duration of less than 15 days), radionuclide therapy at any time, or interferon, chemoembolization or chemotherapy within six months prior to study entry. Full inclusion and exclusion criteria are defined in the primary publication [19].

Patients were randomized (1:1) to receive subcutaneous lanreotide 120 mg or placebo (sodium chloride solution) every four weeks (±1 day) for 96 weeks (maximum of 24 injections). Randomization was conducted using computer-generated randomization lists based on the presence or absence of tumor progression at baseline and on the receipt or non-receipt of previous therapies. As a result of the different appearance of lanreotide and placebo injections, they were prepared and administered by an independent healthcare professional to maintain investigator blinding.

Study visits were scheduled during screening and at weeks one (baseline, first injection visit), 12, 24, 36, 48, 72 and 96. Two computed tomography (CT) scans were performed during screening with an interval of 12 to 24 weeks to determine baseline disease-progression status, and randomization took place within four weeks of the second CT scan.

The CLARINET study was conducted in accordance with the Declaration of Helsinki, the International Conference on Harmonisation Consolidated Guideline on Good Clinical Practice, and local regulatory requirements. Trial documentation was approved by the institutional review board at each study site (Table S2), and all patients provided written informed consent to participate in the study.

### 2.2. Study Assessments and Analyses

The primary endpoint of the CLARINET study was PFS, which was defined as time from randomization to final tumor assessment (scheduled at 96 weeks after first study treatment) to either PD or patient death. Patients who were alive without PD were censored at the date of last visit. PD was defined on the basis of RECIST (v1.0; centrally assessed).

This post hoc analysis evaluated the impact of diabetic status and metformin use in patients with DM on PFS in the whole patient population and in predefined patient subgroups corresponding to study randomization cohorts (i.e., patients treated with lanreotide or placebo). In addition, we investigated the efficacy of lanreotide when compared with placebo in the subgroups of patients with or without DM, and the impact of metformin use on PFS in patients with DM, overall and by treatment group (i.e., lanreotide compared with placebo). Finally, we assessed the independent impact of DM status with regards to other factors potentially affecting the clinical outcomes of advanced NET patients (e.g., baseline characteristics). Adverse events (AEs), recorded at four weekly visits during the study, were also evaluated by diabetic status.

Patients with DM were defined on the basis of at least one of the following criteria at baseline or after treatment initiation: medical diagnosis of type 1 or type 2 DM before or after treatment initiation; prior or ongoing use of antidiabetic drugs; glycated hemoglobin (HbA_1c_) ≥ 6.5%; fasting plasma glucose ≥ 7 mmol/L; or non-fasting plasma glucose ≥ 11.11 mmol/L [20]. Patients were defined as without DM if they did not meet any of these criteria during the course of the study.

### 2.3. Statistical Analyses

These analyses were conducted in patient subsets within the intention-to-treat population, consisting of all randomized patients. The recruitment target, based on the primary endpoint of the CLARINET study, was 200 patients.

PFS was summarized using Kaplan–Meier estimates; *p* values were derived from the log rank test and hazard ratios (HRs) were estimated using a Cox proportional hazards model for PFS. Cox proportional hazards models were used to investigate whether DM status at baseline was significantly associated with patients’ PFS. The models included the baseline stratification factors (PD at baseline {assessed by CT scan within four weeks before starting treatment} and previous therapy), study treatment (lanreotide compared with placebo) and DM status. The interaction of DM status with study treatment was also investigated.

Multivariable Cox models were used to assess the impact of baseline DM on PFS relative to other factors (sex, body mass index {BMI}, hepatic tumor load, tumor type, tumor grade, chromogranin A at baseline).

A sensitivity analysis was performed to exclude a relevant effect deriving from the time-on-treatment bias, i.e., early interruption of lanreotide therapy as a result of disease progression resulting in lower exposure to the drugs relevant to this analysis and a consequently lower incidence of diabetes in poorly responding patients. In this post hoc analysis, patients in the DM group were excluded if DM was diagnosed after the initiation of study treatment.

An additional analysis was also performed to exclude a potential immortal time bias related to metformin intake, i.e., the possibility that patients taking metformin are those who most benefited from lanreotide treatment and consequently were more likely to develop treatment-related DM due to longer treatment exposure. Thus, only patients without disease progression at three months after treatment initiation were included. Patients included in this analysis were then divided into two groups: Group 1, patients taking metformin at three months (including those taking metformin before treatment initiation); Group 2, patients not taking metformin at three months (including those who never took metformin and those who initiated metformin treatment later than three months after treatment initiation).

Differences within treatment groups based on metformin use and between treatment groups stratified by metformin use in the frequency of treatment-emergent AEs (TEAEs) were analyzed using Fisher’s exact tests. TEAEs were defined as any AE that occurred during the active phase of the study if it was not present prior to receiving the first dose of study drug, or it was present prior to receiving the first dose of study drug but the intensity increased during the active phase of the study.

### 2.4. Study Data

Statistical analyses were performed by an external CRO (INC Research), managed by the Sponsor’s Biometry Department using Statistical Analysis System (SAS)^®^ version 9.3 (SAS Institute Inc., Cary, NC, USA, 2004). All tables, listings and figures were also produced, using SAS^®^ version 9.3. All authors had access to the study data and reviewed and approved the final manuscript.

## 3. Results

### 3.1. Patients

**Figure 1:**
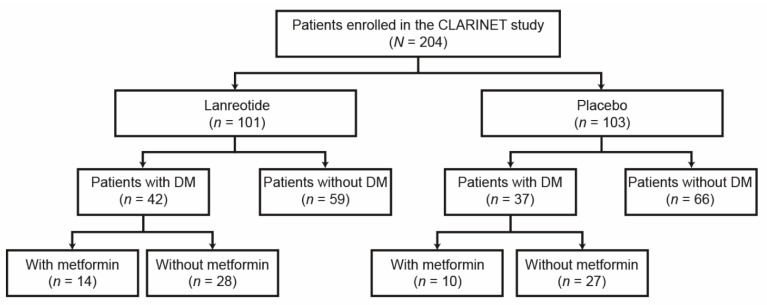
Patient flow diagram. Further details of patient disposition from the CLARINET study are reported in Caplin et al., 2014 [19]. DM, diabetes mellitus.

A total of 204 patients enrolled in the CLARINET study were randomized to receive lanreotide (*n =* 101) or placebo (*n =* 103). As shown as a patient flow diagram in Figure 1, of the enrolled patients, 79 had DM, while 125 did not have DM. Of the 79 patients with DM, 23 developed DM after treatment initiation (lanreotide, *n =* 17; placebo, *n =* 6). In addition, 24 patients with DM received metformin as a concomitant medication during the course of the study (lanreotide group, *n =* 14; placebo group, *n =* 10), while 11 patients received metformin later than three months after treatment initiation.

Baseline patient characteristics by DM status and treatment group are shown in Table 1. Mean (standard deviation {SD}) age was similar between patients with DM and patients without DM (62.4 {11.5} years compared to 63.3 {8.7} years, respectively; *p* = 0.679). Compared with patients without DM, a higher proportion of patients with DM were male (44.0% compared with 65.8%, respectively; *p* = 0.002) and had primary tumors in the pancreas (33.6% compared with 62.0%, respectively; *p* = 0.001) (Table 1). Among patients randomized to receive lanreotide, a significantly higher proportion with DM had received previous therapy for non-functioning enteropancreatic NETs at study entry when compared with patients without DM (26.2% compared with 8.5%; *p* = 0.016). As would be expected, patients with DM also had a significantly higher mean BMI, higher mean HbA_1c_ and higher mean fasting glucose compared with patients without DM (Table 1). Baseline characteristics of patients with DM by metformin use are presented in Table S1.

### 3.2. Effects of DM and Study Treatment on PFS

**Figure 2:**
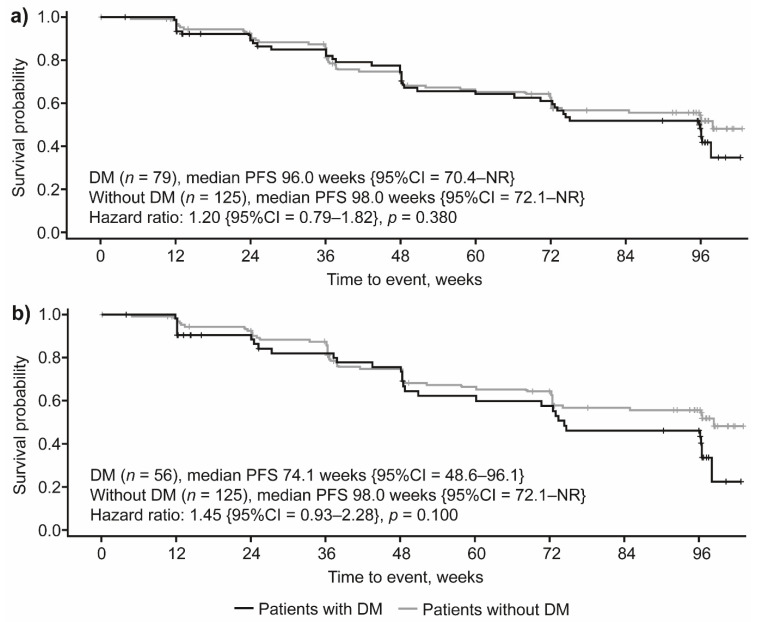
PFS by DM status in (**a**) all patients and (**b**) excluding patients diagnosed with DM after study treatment initiation. CI, confidence interval; DM, diabetes mellitus; NR, not reached; PFS, progression-free survival.

When considering the whole population of patients evaluated, we did not find statistically significant differences in PFS between patients with or without DM (median PFS of 96.0 weeks compared to 98.0 weeks, respectively; HR 1.20 {95% confidence interval {CI} 0.79–1.82}; *p* = 0.380) (Figure 2a). In the subset of patients with DM diagnosed prior to study initiation, the median PFS was 74.1 weeks (HR 1.45 {95%CI = 0.93–2.28}; *p* = 0.100) (Figure 2b).

**Figure 3:**
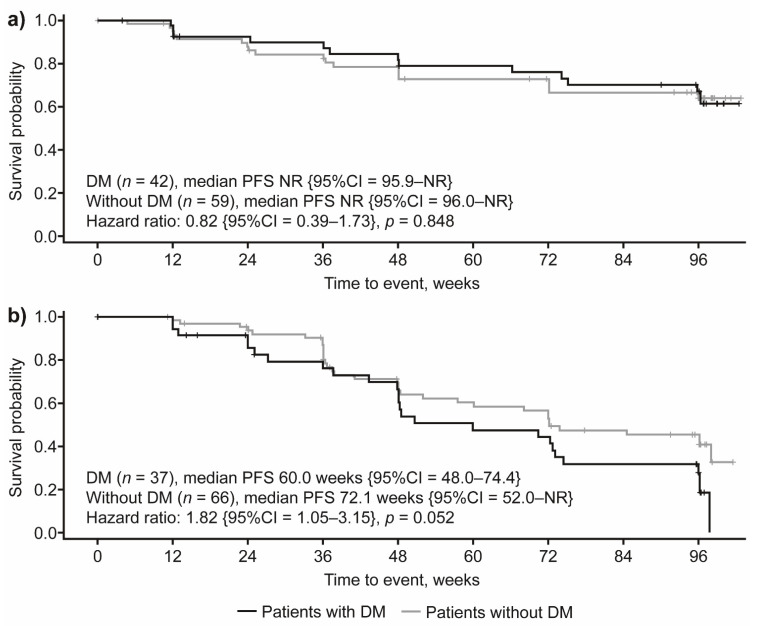
PFS by DM status in patients receiving (**a**) lanreotide 120 mg and (**b**) placebo. CI, confidence interval; DM, diabetes mellitus; NR, not reached; PFS, progression-free survival.

In patients treated with lanreotide, median PFS was not reached regardless of DM status, and no significant PFS differences were observed based on DM status (HR: 0.82 {95%CI = 0.39–1.73}; *p* = 0.848; Figure 3a). Conversely, in the placebo arm there was a trend towards lower PFS in patients with DM when compared with those without DM (median PFS: 60.0 weeks and 72.1 weeks, respectively; HR: 1.82 {95%CI = 1.05–3.15}; *p* = 0.052; Figure 3b).

When compared with placebo, lanreotide treatment was associated with longer PFS in patients with DM (HR: 0.27 {95%CI = 0.13–0.57}; *p* < 0.001; Figure S1A). In patients without DM, although PFS was statistically significantly longer in patients receiving lanreotide compared with placebo (HR: 0.64 {95%CI = 0.35–1.15}; *p* = 0.038), the Kaplan–Meier plots for lanreotide treatment and placebo crossed, indicating non-proportional hazards, which does not allow superiority to be concluded in this patient subgroup (Figure S1B). Additionally, although the relative PFS benefit associated with lanreotide was higher in patients with DM compared with patients without DM (as evaluated through HR of disease progression), the interaction between DM status and study treatment on PFS was not statistically significant (HR: 0.41 {95%CI = 0.16–1.05}; *p* = 0.063).

### 3.3. Impact of Metformin Use on PFS of Patients with DM

In patients diagnosed with DM prior to treatment initiation who received lanreotide, median PFS was not reached in those who were not receiving concomitant metformin, while median PFS was reached at 95.9 weeks in patients receiving metformin; however, this difference did not reach statistical significance (HR: 4.27 {95% CI 0.20–92.62}; *p* = 0.402; Figure S2A). In the placebo group, median PFS was significantly (97%) longer in patients taking metformin when compared with patients not receiving concomitant metformin (97.7 weeks compared with 50.7 weeks, respectively; HR: 0.03 {95%CI = 0.00–0.63}; *p* = 0.009; Figure S2B).

**Figure 4:**
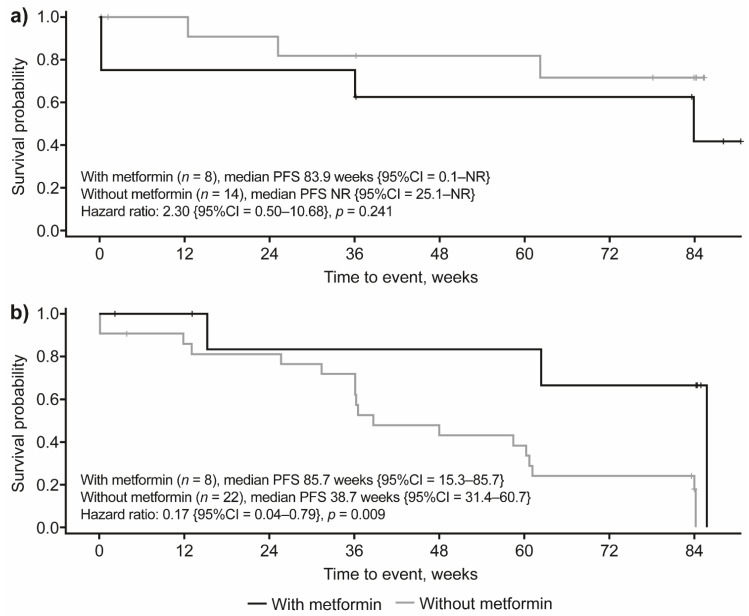
PFS by metformin treatment in patients with DM who developed DM prior to study treatment in patients receiving (**a**) lanreotide 120 mg and (**b**) placebo (additional analysis). The additional analysis included patients without disease progression at three months after treatment initiation. Based on this criterion, four patients were excluded from this analysis (lanreotide group, *n =* 3; placebo group, *n =* 1). CI, confidence interval; DM, diabetes mellitus; NR, not reached; PFS, progression-free survival.

To confirm these results, an additional analysis was performed that excluded patients experiencing disease progression during the first three months on treatment. This analysis showed that, among patients with a diagnosis of DM prior to lanreotide initiation (i.e., experimental arm), median PFS was not reached in patients not receiving concomitant metformin, while for patients receiving metformin, median PFS was reached at 83.9 weeks; however, this difference was not statistically significant (HR: 2.30 {95%CI = 0.5–10.68}; *p* = 0.241; Figure 4a). In the placebo group, median PFS was significantly (83%) longer in patients with DM taking metformin compared to patients with DM not receiving concomitant metformin (85.7 weeks compared with 38.7 weeks, respectively; HR: 0.17 {95%CI = 0.04–0.79}; *p* = 0.009; Figure 4b).

### 3.4. Impact of DM Status and Concomitant Metformin Use on PFS by Study Treatment

**Figure 5:**
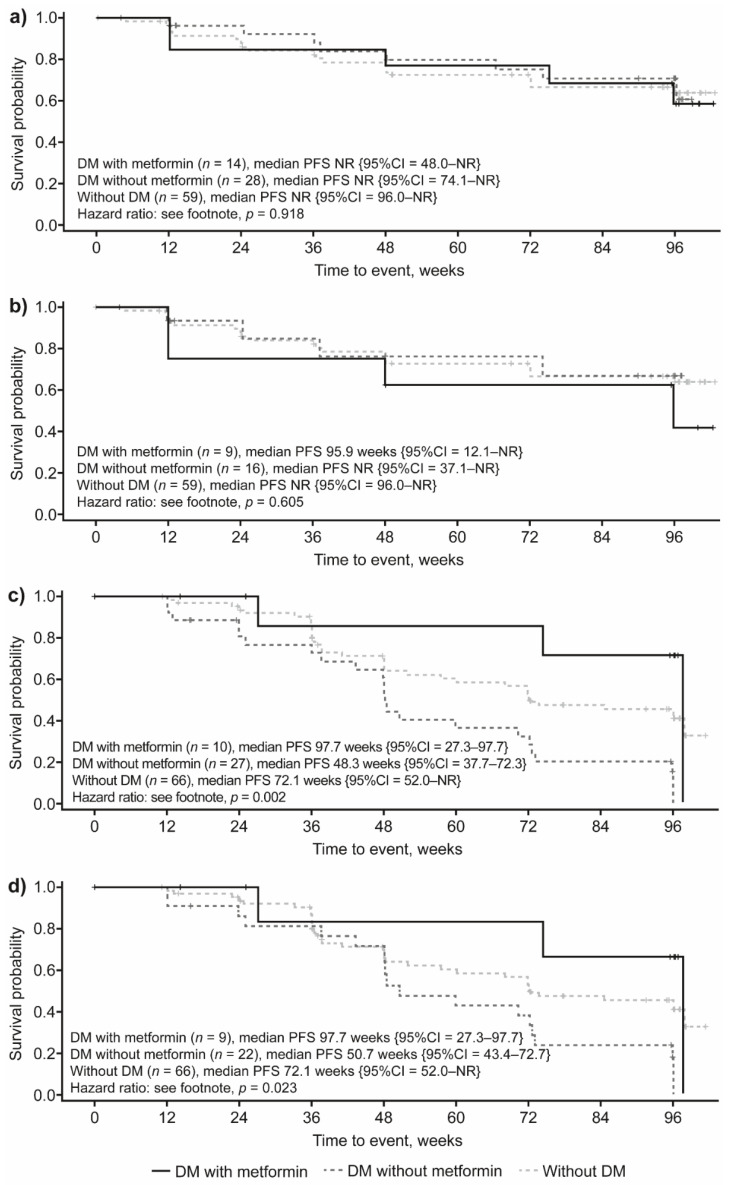
PFS by DM status and metformin treatment in (**a**) all patients receiving lanreotide 120 mg, (**b**) patients receiving lanreotide 120 mg, excluding those who developed DM after the start of treatment, (**c**) all patients receiving placebo and (**d**) patients receiving placebo, excluding those who developed DM after the start of treatment. HR: (**a**) DM with metformin compared with without DM: 0.94 {95%CI = 0.32; 2.77}; DM with metformin compared with DM without metformin: 1.22 {95%CI = 0.38; 3.87}; DM without metformin compared with without DM: 0.77 {95%CI = 0.33; 1.80}; (**b**) DM with metformin compared with without DM: 0.59 {95%CI = 0.18; 1.97}; DM with metformin compared with DM without metformin: 0.24 {95%CI = 0.07; 0.80}; DM without metformin compared with without DM: 2.50 {95%CI = 1.41; 4.44}; (**c**) DM with metformin compared with without DM: 1.23 {95%CI = 0.37; 4.05}; DM with metformin compared with DM without metformin: 1.81 {95%CI = 0.43; 7.71}; DM without metformin compared with without DM: 0.68 {95%CI = 0.22; 2.05}; (**d**) DM with metformin compared with without DM: 0.66 {95%CI = 0.20; 2.19}; DM with metformin compared with DM without metformin: 0.31 {95%CI = 0.09; 1.05}; DM without metformin compared with without DM: 2.15 {95%CI = 1.18; 3.92}. CI, confidence interval; DM, diabetes mellitus; HR, hazard ratio; NR, not reached; PFS, progression-free survival.

In patients receiving lanreotide, median PFS was not reached during the study regardless of DM status or metformin use (*p* = 0.918; Figure 5a). When only patients who developed DM prior to lanreotide initiation were included in the analysis, median PFS was still not reached in patients with DM not taking metformin or in patients without DM, while median PFS was reached at 95.9 weeks in patients with DM receiving concomitant metformin; however, these differences did not reach statistical significance (*p* = 0.605; Figure 5b). In the subgroup of patients with DM randomized to the placebo arm, patients receiving concomitant metformin had significantly longer PFS (97.7 weeks) when compared with patients not receiving metformin (48.3 weeks), as well as with patients without DM (72.1 weeks; *p* = 0.002; Figure 5c). Similar results were observed when only patients who developed DM prior to treatment initiation were included among patients with DM in this analysis (*p* = 0.023; Figure 5d).

### 3.5. Multivariable Analysis in Patients Who Had Developed DM Prior to Study Treatment Initiation

To assess the independent impact of DM status on PFS, we fitted a multivariable model to adjust its impact for the following covariates: PD at baseline (i.e., before treatment initiation); previous therapies received; DM status; and interaction between DM and treatment (i.e., lanreotide compared with placebo). In this model, lanreotide was associated with significantly lower risk of disease progression (HR: 0.53 {95%CI = 0.31–0.89}; *p* = 0.017), while PD at baseline was associated with a significantly worse PFS (HR: 3.19 {95%CI = 1.34–7.61}; *p* = 0.009). On the other hand, neither baseline DM nor the interaction term between lanreotide treatment and baseline DM showed a significant association with PFS (HR: 1.64 {95%CI = 0.95–2.84}; *p* = 0.079 and HR: 0.78 {95%CI = 0.29–2.09}; *p* = 0.619, respectively). Furthermore, previous therapy at baseline was not significantly associated with PFS (HR: 1.56 {95%CI = 0.88–2.77}; *p* = 0.130) (Table 2).

We also fitted another stepwise multivariable model in three stages that included in stage 1 all the covariates found to be potentially relevant clinical factors, which were identified on the basis of a univariable analysis, by using 10% as a significance level from the original study analysis. The baseline covariates included in the first model were as follows: treatment received, PD at baseline, previous therapy, sex, BMI, hepatic tumor load, primary tumor type, tumor grade, chromogranin A levels, DM, metformin use, and insulin use. In this first model, BMI, hepatic tumor load and primary tumor type were statistically significant (at *p* < 0.10). Baseline DM did not show a statistically significant association with PFS and was not investigated further (HR: 1.01 {95%CI = 0.50–2.04}; *p* = 0.969). In stage 2, all previous significant covariates as well as their interaction with treatment were included in another multivariable analysis. None of the interactions were statistically significant. Finally, in stage 3, only significant covariates from stage 1 and stage 2 were included. In this multivariable model, lanreotide maintained a significant association with better PFS compared to placebo (HR: 0.41 {95%CI = 0.24–0.67}; *p* < 0.001). Other covariates independently associated with PFS were: higher hepatic tumor load (HR: 2.33 {95%CI = 1.09–4.98}, *p* = 0.002), primary tumor type (HR: 0.42 {95%CI = 0.19–0.96}; *p* = 0.018) and BMI (HR: 0.58 {95%CI = 0.36–0.94}; *p* = 0.028) (Table 3).

### 3.6. Safety

A summary of TEAEs is presented in Table 4. In patients with DM, the frequency of any TEAEs was similar, with no significant difference within treatment groups based on metformin use (lanreotide: with metformin, 100%; without metformin, 92.9% {*p* = 0.545}; placebo: with metformin, 90%; without metformin, 88.9% {*p* = 1.000}) or between treatment groups (lanreotide compared to placebo with metformin {*p* = 0.417}; lanreotide compared to placebo without metformin {*p* = 0.670}). In these patient groups, diarrhea was the most frequently reported TEAE, with the frequency being significantly higher in the lanreotide with metformin subgroup (64.3%) compared with the lanreotide without metformin subgroup (28.6%; *p* = 0.045); no significant differences were observed between the placebo with metformin compared with placebo without metformin subgroups, or between the lanreotide and placebo treatment groups. The frequency of injection site pain (*p* = 0.011) and DM (*p* = 0.031) were also found to be significantly higher in the lanreotide with metformin subgroup compared with the lanreotide without metformin subgroup, although it should be noted that the sample size is small (Table 4).

In patients without DM, those receiving lanreotide tended to have a lower frequency of any TEAEs compared with placebo (83.1% compared to 90.9%, respectively), although differences were not found to be statistically significant (*p* = 0.283). Diarrhea was still the most frequent TEAE, with similar incidence in both lanreotide and placebo groups (30.5% and 34.8%, respectively; *p* = 0.704) (Table 2).

Further details of the safety results for the overall population have been published in the CLARINET study primary manuscript [19].

## 4. Discussion

In this post hoc analysis of the CLARINET study, we found that lanreotide 120 mg in patients with advanced, non-functional enteropancreatic NETs is associated with better PFS when compared with placebo regardless of DM status. In particular, the presence of DM at baseline did not negatively affect PFS in the final multivariable model, as the interaction between DM status (yes compared to no) and the type of treatment (lanreotide compared to placebo) on PFS was not statistically significant (*p* = 0.079). These data suggest that DM might not impact the prognosis of patients with advanced enteropancreatic NETs.

However, in univariable analysis, lanreotide compared with placebo did appear to provide greater benefit to patients with DM compared to patients without DM (HR: 0.27; *p* < 0.001 and HR: 0.64; *p* = 0.038, respectively). Although multivariable analysis failed to reveal a significant association between the interaction of baseline DM and the treatment on PFS (possibly also as a result of insufficient statistical power of our post hoc analysis), we still believe that this finding is of potential interest. Indeed, patients with type 2 DM typically have high blood glucose, insulin and IGF1 levels, as NET cell proliferation in patients with DM could at least in part depend on extracellular growth factor-mediated stimulation of the insulin-receptor/IGF1R/PI3K/Akt/mTORC1 pathway [21]. It is thought that lanreotide could reverse the effects of high blood insulin/IGF1 concentration though activating the somatostatin receptor and inhibiting the PI3K/Akt/mTORC1 pathway in cancer cells [21]. The antisecretive effects of lanreotide on pancreatic beta-cells, which may result in reduced plasmatic insulin secretion and IGF1 production, could further contribute to the observed benefit of lanreotide in patients with DM [22,23].

Another interesting result of this post hoc analysis is that patients with DM randomized to the placebo group had statistically significantly lower PFS when compared with patients without DM (*p* = 0.052), whereas DM was not associated with PFS differences in patients randomized to lanreotide (*p* = 0.848). Collectively considered, these data indicate that DM could be associated with a more aggressive disease course in the absence of an active anti-NET treatment. On the other hand, DM might lose its negative prognostic role when an effective antitumor therapy, such as lanreotide, is administered. In a real-life clinical setting, most patients with advanced well- or moderately differentiated NETs receive an active antitumor treatment; therefore, DM is unlikely to have a clinically relevant impact in this patient population.

Our post hoc analysis also showed a potentially favorable impact of metformin use on the PFS of patients with advanced, non-functional enteropancreatic NETs who developed DM prior to study treatment. This effect was limited to patients randomized to the placebo arm, where median PFS in patients using metformin had more than doubled compared to patients not receiving metformin (*p* = 0.009). When patients were stratified according to diabetic status and metformin use, patients with DM randomized to the placebo group and receiving concomitant metformin had significantly longer PFS compared to patients not receiving metformin (*p* = 0.002). While these results need to be prospectively validated, they may provide the first evidence that metformin could have some antitumor activity when used as a single-agent therapy in patients with more indolent enteropancreatic NETs. In particular, these results are consistent with previous preclinical and retrospective studies [5,8,9], and may provide the first prospective evidence to suggest that metformin might contribute to halt NET cell growth and proliferation. The mechanism of metformin is thought to be through activation of AMPK and subsequent inhibition of mTORC1, which plays a crucial role in the pathogenesis and progression of these neoplasms [8,9]. Conversely, in the subgroup of patients with DM who were randomized to receive lanreotide, no significant association was observed between metformin use and PFS (*p* = 0.241). Since median PFS was not reached in lanreotide-treated patients and the number of patients with DM receiving metformin was low, the impact of DM and metformin use could not be independently assessed. One potential explanation is that lanreotide alone could be sufficiently potent to inhibit the mTORC1 axis in patients with indolent disease, such as those patients enrolled in the CLARINET trial.

Our results contrast with the results of the PRIME-NET multicenter, retrospective study in patients with advanced panNETs, in which patients with DM taking metformin had significantly longer PFS when compared with patients receiving other antidiabetic therapies [5]. These discrepancies could in part be explained by differences in study design, as the PRIME-NET study included patients with PD at enrollment, with well-differentiated G1-G2 pancreatic tumors (associated with an aggressive clinical course), and patients received more active antitumor therapies (i.e., somatostatin analogs with or without everolimus), while in the CLARINET study patients had stable disease at enrollment, tumors of different primary origin (including panNETs and intestinal NETS with a Ki67 ≤ 10%), and received single-agent treatment with lanreotide or placebo. The PRIME-NET study was also a large, retrospective, Italian study, while CLARINET was an international phase III trial in which randomization arms were stratified by diabetic status, among other factors. Based on these crucial differences in the design of these two studies, a different impact of metformin on the prognosis of patients could be expected. In particular, in patients with more aggressive neoplasms that are associated with a worse prognosis (e.g., advanced, progressive panNETs), metformin could produce additive or synergistic antitumor effects when used in combination with somatostatin analogs with or without everolimus, thus improving the efficacy of standard systemic treatments. Conversely, metformin may provide no clinical benefit when used in patients with less aggressive neoplasms receiving an active antitumor therapy, such as lanreotide, while the antitumor activity of metformin in this context could become relevant when it is used as a single therapy, i.e., when no active treatments that inhibit the mTORC1 axis are used.

Safety analyses were consistent with findings of the primary analysis, with diarrhea being the most common TEAE [19]. Although the frequency of diarrhea and injection site pain were found to be significantly higher in the lanreotide with metformin subgroup compared with the lanreotide without metformin subgroup, these results should be interpreted with caution as the sample sizes were small.

This post hoc analysis of the CLARINET study has several limitations that must be considered when interpreting these results. The low number of patients with DM taking metformin prevented a formal analysis of any potential synergistic effect of this drug with lanreotide. In addition, as this was a post hoc analysis, subgroups were not stratified to account for patient’s baseline characteristics. For instance, a significantly higher proportion of patients with DM had previously received therapy for non-functional enteropancreatic NETs compared with those without DM, which could potentially be the result of a better prognosis in patients without DM in the placebo group. Furthermore, a statistically significant difference was noted in primary tumor location between patient groups, which may have affected the study results, as patient prognosis differs dependent on primary tumor location (e.g., worse for panNET compared with midgut). Despite these limitations, taken together, the results of the PRIME-NET study and the presented CLARINET post hoc analysis indicate that patients with DM with more aggressive advanced NETs could benefit from adding metformin to the standard-of-care therapy with somatostatin analogs. Patients with less aggressive NETs, such as those enrolled in the CLARINET trial, could potentially benefit from metformin even in the absence of an active antitumor therapy. This hypothesis is supported by the finding that placebo-treated patients with DM taking metformin had similar PFS when compared with patients with DM receiving active treatment (with or without metformin). Although provocative, this conclusion is limited by the low number of patients considered for subgroup analysis and will require further validation in future prospective trials.

Two ongoing clinical trials, conducted by the Fondazione IRCCS Istituto Nazionale Tumori (ENETS Center of Excellence), Milan, Italy, are currently investigating the safety and antitumor activity of adding metformin to standard antitumor therapies in patients with advanced NETs. The first study (MetNet1, NCT02294006), is a prospective, open-label, single-arm trial in which patients with advanced panNETs will receive metformin in combination with first-line somatostatin analogs and everolimus, and the second (MetNet2, NCT02823691) is a pilot, single-arm, open-label, prospective study investigating the safety profile of upfront metformin in combination with lanreotide in patients with advanced well-differentiated gastrointestinal and lung NETs [24]. In both studies, patients without DM taking metformin are included, based on published preclinical data indicating that metformin also produces direct (cell-autonomous) antitumor effects, independent of glucose extracellular concentration [25,26].

## 5. Conclusions

The present post hoc analysis of the CLARINET trial demonstrated that, compared with placebo, lanreotide was more effective at prolonging PFS in patients both with and without DM with advanced, non-functional enteropancreatic NETs. DM did not emerge as a negative prognostic factor in this patient population, and although the lanreotide-DM interaction was not statistically significant, the HR for PFS between patient subgroups suggests that lanreotide might be associated with improved relative PFS benefit in patients with DM when compared with patients without DM. Finally, metformin use in placebo group patients with enteropancreatic NETs who have diabetes may be associated with improved PFS. Ongoing prospective studies will clarify the safety and antitumor activity of metformin in combination with lanreotide.

## Figures and Tables

**Table 1:** Baseline characteristics by DM status (intention-to-treat population).

Characteristic	with DM			without DM		
	Lanreotide	Placebo	Overall	Lanreotide	Placebo	Overall
	(*N =* 42)	(*N =* 37)	(*N =* 79)	(*N =* 59)	(*N =* 66)	(*N =* 125)
Sex, *n* (%)	*n =* 42	*n =* 37	*n =* 79	*n =* 59	*n =* 66	*n =* 125
Male	25 (59.5)	27 (73.0) ^‡^	52 (65.8) ^‡^	28 (47.5)	27 (40.9) ^‡^	55 (44.0) ^‡^
Female	17 (59.5)	10 (27.0)	27 (34.2)	31 (52.5)	39 (59.1)	70 (56.0)
Mean age (SD), in years	*n =* 42	*n =* 37	*n =* 79	*n =* 59	*n =* 66	*n =* 125
	62.9 (9.2)	63.7 (8.1)	63.3 (8.7)	63.6 (10.2)	61.3 (12.5)	62.4 (11.5)
BMI, kg/m^2^ (SD)	*n =* 39	*n =* 36	*n =* 75	*n =* 57	*n =* 63	*n =* 120
	29.7 (5.2) *	28.0 (6.1)	28.9 (5.7) *	25.7 (3.9) *	25.9 (5.7)	25.8 (4.9) *
WHO performance status, *n* (%)	*n =* 42	*n =* 37	*n =* 79	*n =* 59	*n =* 66	*n =* 125
0 (normal activity)	36 (85.7)	30 (81.1)	66 (83.5)	48 (81.4)	54 (81.8)	102 (81.6)
1 (restricted activity)	6 (14.3)	6 (16.2)	12 (15.2)	11 (18.6)	11 (16.7)	22 (17.6)
2 (in bed <50% of the time)	0 (0.0)	1 (2.7)	1 (1.3)	0 (0.0)	1 (1.5)	1 (0.8)
Tumor grade, *n* (%) ^a^	*n =* 42	*n =* 37	*n =* 79	*n =* 59	*n =* 64	*n =* 123
Grade 1	25 (59.5)	24 (64.9)	49 (62.0)	44 (74.6)	48 (75.0)	92 (74.8)
Grade 2	17 (40.5)	13 (35.1)	30 (38.0)	15 (25.4)	16 (25.0)	31 (25.2)
Primary tumor location, *n* (%)	*n =* 42 ^‡^	*n =* 37 ^‡^	*n =* 79 ^†^	*n =* 59 ^‡^	*n =* 66 ^‡^	*n =* 125 ^†^
Pancreas	24 (57.1)	25 (67.6)	49 (62.0)	18 (30.5)	24 (36.4)	42 (33.6)
Midgut	8 (19.0)	8 (21.6)	16 (20.3)	25 (42.4)	32 (48.5)	57 (45.6)
Hindgut	4 (9.5)	1 (2.7)	5 (6.3)	7 (11.9)	2 (3.0)	9 (7.2)
Other/unknown	6 (14.3)	3 (8.1)	9 (11.4)	9 (15.3)	8 (12.1)	17 (13.6)
Previous therapy for non-functional enteropancreatic NETs at study entry, *n* (%)	*n =* 42	*n =* 37	*n =* 79	*n =* 59	*n =* 66	*n =* 125
	11 (26.2) ^‡^	3 (8.1)	14 (17.7)	5 (8.5) ^‡^	13 (19.7)	18 (14.4)
Previous surgery of primary tumor, *n* (%)	*n =* 42	*n =* 37	*n =* 79	*n =* 59	*n =* 66	*n =* 125
	17 (40.5)	12 (32.4)	29 (36.7)	23 (39.0)	27 (40.9)	50 (40.0)
Hepatic tumor load, *n* (%)	*n =* 42	*n =* 37	*n =* 79 ^‡^	*n =* 59	*n =* 66	*n =* 125 ^‡^
≤25%	23 (54.8)	23 (62.2)	46 (58.2)	39 (66.1)	52 (78.8)	91 (72.8)
>25%	19 (45.2)	14 (37.8)	33 (41.8)	20 (33.9)	14 (21.2)	34 (27.2)
Mean HbA_1c_ (SD), %	*n =* 40	*n =* 37	*n =* 77	*n =* 59	*n =* 65	*n =* 124
	6.9 (1.5) *	6.8 (1.2) *	6.9 (1.3) *	5.6 (0.4) *	5.6 (0.3) *	5.6 (0.3) *
Median (range) fasting glucose, mmol/L	*n =* 32	*n =* 30	*n =* 62	*n =* 50	*n =* 48	*n =* 98
	6.5 (3.6; 11.6) *	6.5 (4.6; 14.5) *	6.5 (3.6; 14.5) *	4.9 (4.1; 6.9) *	4.8 (3.4; 6.1) *	4.8 (3.4; 6.9) *
Median (range) non-fasting glucose, mmol/L	*n =* 6	*n =* 5	*n =* 11	*n =* 7	*n =* 16	*n =* 23
	7.1 (3.2; 22.6)	5.8 (3.3; 9.8)	6.8 (3.2; 22.6)	5.5 (4.6; 5.9)	4.8 (3.9; 7.8)	4.9 (3.9; 7.8)

BMI, body mass index; DM, diabetes mellitus; HbA_1c_, glycated hemoglobin; HPF, high power fields; Ki67, proliferation index; NETs, neuroendocrine tumors; SD, standard deviation; WHO, World Health Organization. * *p* < 0.001, ^†^ *p* < 0.01, and ^‡^ *p* < 0.05 between patients with DM and patients without DM. A Wilcoxon–Mann–Whitney test was used to analyze quantitative variables, and Pearson’s Chi-square test to analyze qualitative variables. ^a^ Grade 1 = mitotic count <2 mitoses/10 HPF and/or Ki67 ≤2%; Grade 2 = mitotic count 2 to 20 mitoses/10 HPF and/or Ki67 >2% to 10%.

**Table 2:** Exploratory analysis to investigate the influence of DM status at baseline covariate on PFS (intention-to-treat population).

Variable (Reference Level)	HR (95% CI)	*p* Value
Diabetes at baseline (No)	1.64 (0.95 to 2.84)	0.079
Previous therapy at entry (No)	1.56 (0.88 to 2.77)	0.130
Progression at baseline (No)	3.19 (1.34 to 7.61)	0.009
Treatment (Placebo)	0.53 (0.31 to 0.89)	0.017
Interaction between treatment and DM at baseline interaction (No)	0.78 (0.29 to 2.09)	0.619

CI, confidence interval; DM, diabetes mellitus; HR, hazard ratio; PFS, progression-free survival. Each baseline covariate was included one at a time in a Cox proportional hazards model (treatment and the baseline stratification factors centrally assessed progression at baseline and previous therapy for non-functional-NET at entry are forced into all models). Any factors found to be significant at *p* < 0.10 (via the Wald Chi-square test statistic) were considered potentially important.

**Table 3:** Exploratory analysis into the influence of baseline covariates on PFS time, final model (intention-to-treat population).

Variable (Reference Level)	HR (95% CI)	*p* Value (Probability > Chi-Squared)
Treatment (Placebo)	0.41 (0.24 to 0.67)	<0.001
Progression at baseline (No)	4.62 (1.68 to 12.74)	0.003
Previous therapy at entry (No)	1.42 (0.76 to 2.65)	0.275
BMI (≤ median value)	0.58 (0.36 to 0.94)	0.028
Hepatic tumor load: >0%, ≤10% (0%)	0.88 (0.44 to 1.76)	–
Hepatic tumor load: >10%, ≤25% (0%)	1.17 (0.54 to 2.52)	–
Hepatic tumor load: >25%, ≤50% (0%)	3.17 (1.52 to 6.62)	–
Hepatic tumor load: >50% (0%)	2.33 (1.09 to 4.98)	0.002
Primary tumor type: Mid Gut (Pancreas)	0.48 (0.28 to 0.82)	–
Primary tumor type: Hind Gut (Pancreas)	1.17 (0.47 to 2.93)	–
Primary tumor type: Other/Unknown (Pancreas)	0.42 (0.19 to 0.96)	0.018

BMI, body mass index; CI, confidence interval; DM, diabetes mellitus; HR, hazard ratio; PFS, progression-free survival. This table presents the final stage of three stages of modelling. Stage 1 combined all the covariates found to be potentially influential in a previous analysis into a multivariable model to investigate which covariates are still important in the presence of the other terms (at a significance level of 10%); Stage 2 investigated the role of the interaction with treatment terms (at a significance level of 10%); Stage 3 then provided the data for the final model. Model is based on the intention-to-treat population excluding those patients who developed DM after baseline (*n* = 181); 13 patients with missing values in stage 1, seven patients with missing values in Stage 2 and Stage 3.

**Table 4:** Summary of TEAEs.

Type of TEAE	Patients with DM				Patients without DM	
	Lanreotide		Placebo		Lanreotide (*N =* 59) *n* (%) {m}	Placebo (*N =* 66) *n* (%) {m}
	with Metformin (*N =* 14) *n* (%) {m}	without Metformin (*N =* 28) *n* (%) {m}	with Metformin (*N =* 10) *n* (%) {m}	without Metformin (*N =* 27) *n* (%) {m}		
Any TEAE	14 (100) {191}	26 (92.9) {187}	9 (90.0) {109}	24 (88.9) {206}	49 (83.1) {479}	60 (90.9) {500}
Diarrhea	9 (64.3) {16} *	8 (28.6) {12} *	3 (30.0) {5}	10 (37) {16}	18 (30.5) {29}	23 (34.8) {55}
Abdominal pain	4 (28.6) {6}	6 (21.4) {9}	1 (10.0) {1}	5 (18.5) {6}	14 (23.7) {17}	11 (16.7) {25}
Vomiting	4 (28.6) {4}	8 (28.6) {10}	0	2 (7.4) {2}	7 (11.9) {10}	7 (10.6) {25}
Constipation	1 (7.1) {2}	5 (17.9) {5}	1 (10.0) {1}	4 (14.8) {4}	6 (10.2) {7}	8 (12.1) {10}
Flatulence	4 (28.6) {4}	4 (14.3) {4}	0	2 (7.4) {2}	4 (6.8) {5}	7 (10.6) {10}
Nausea	2 (14.3) {10}	5 (17.9) {6}	0	4 (14.8) {4}	7 (11.9) {12}	10 (15.2) {19}
Fatigue	2 (14.3) {3}	2 (7.1) {2}	3 (30.0) {3}	4 (14.8) {5}	6 (10.2) {9}	8 (12.1) {9}
Injection site pain	5 (35.7) {7} *	1 (3.6) {1} *	1 (10.0) {5}	1 (3.7) {1}	2 (3.4) {22}	2 (3) {4}
Nasopharyngitis	2 (14.3) {2}	1 (3.6) {1}	3 (30.0) {3}	3 (11.1) {5}	6 (10.2) {6}	10 (15.2) {14}
Back pain	2 (14.3) {3}	2 (7.1) {2}	1 (10.0) {1}	0	8 (13.6) {8}	10 (15.2) {10}
Arthralgia	2 (14.3) {3}	2 (7.1) {2}	0	3 (11.1) {3}	6 (10.2) {9}	6 (9.1) {7}
Headache	2 (14.3) {2}	3 (10.7) {3}	0	3 (11.1) {4}	11 (18.6) {14}	8 (12.1) {14}
Decreased appetite	4 (28.6) {4}	3 (10.7) {3}	1 (10.0) {1}	4 (14.8) {5}	3 (5.1) {4}	4 (6.1) {5}
DM	5 (35.7) {6} *	2 (7.1) {2} *	3 (30.0) {3}	1 (3.7) {1}	-	-
Dizziness	4 (28.6) {5}	1 (3.6) {1}	1 (10.0) {1}	0	4 (6.8) {6}	1 (1.5) {1}
Weight decreased	3 (21.4) {3}	2 (7.1) {2}	1 (10.0) {1}	2 (7.4) {3}	3 (5.1) {3}	6 (9.1) {6}
Hypertension	5 (35.7) {6}	3 (10.7) {3}	2 (20.0) {2}	0	5 (8.5) {7}	3 (4.5) {3}
Cholelithiasis	3 (21.4) {3}	3 (10.7) {3}	1 (10.0) {1}	2 (7.4) {2}	8 (13.6) {9}	4 (6.1) {4}

DM, diabetes mellitus; m, number of events; TEAE, treatment-emergent adverse event. TEAEs were defined as any AE that occurred during the active phase of the study if it was not present prior to receiving the first dose of study drug, or it was present prior to receiving the first dose of study drug but the intensity increased during the active phase of the study. TEAEs presented are those which occurred in ≥15% of patients with DM receiving active treatment who were taking metformin; ≥15% of with DM receiving active treatment who were not taking metformin; and/or ≥10% of patients without DM receiving active treatment. Differences between groups were analyzed using exact Fisher tests. * *p* < 0.05.

## Data Availability

Where patient data can be anonymized, Ipsen will share all individual participant data that underlie the results reported in this article with qualified researchers who provide a valid research question. Study documents, such as the study protocol and clinical study report, are not always available. Proposals should be submitted to DataSharing@Ipsen.com and will be assessed by a scientific review board. Data are available beginning 6 months and ending 5 years after publication; after this time, only raw data may be available.

## Supplementary Materials

The following are available online at www.mdpi.com/article/10.3390/cancers14010069/s1, Figure S1: PFS by treatment group in patients (**A**) with DM and (**B**) without DM, Figure S2: PFS by metformin treatment in all patients with DM who developed DM prior to study treatment and who received (**A**) lanreotide 120 mg and (**B**) placebo, Table S1: Baseline characteristics of patients with DM by metformin use (intention-to-treat population), Table S2: List of Ethics Committees and/or Institutional Review Boards.
