# Supplementary material for: Impact of Diabetes and Metformin Use on Enteropancreatic Neuroendocrine Tumors: Post Hoc Analysis of the CLARINET Study
Source: Cancers (Basel). 2021 Dec 23;14(1):69. doi: 10.3390/cancers14010069 (PMC8750688; doi:10.3390/cancers14010069)
Supplement: Supplementary file 1 [file cancers-14-00069-s001.zip › cancers-1473791-supplementary.pdf]

cancers MDPI

Supplementary material

Impact of Diabetes and Metformin Use on Enteropancreatic Neuroendocrine Tumors: *Post Hoc* Analysis of the CLARINET Study

Sara Pusceddu, Claudio Vernieri, Massimo Di Maio, Natalie Prinzi, Martina Torchio, Francesca Corti, Jorgelina Coppa, Roberto Buzzoni, Maria Di Bartolomeo, Massimo Milione, Benjamin Regnault, Xuan-Mai Truong Thanh, Vincenzo Mazzaferro and Filippo de Braud

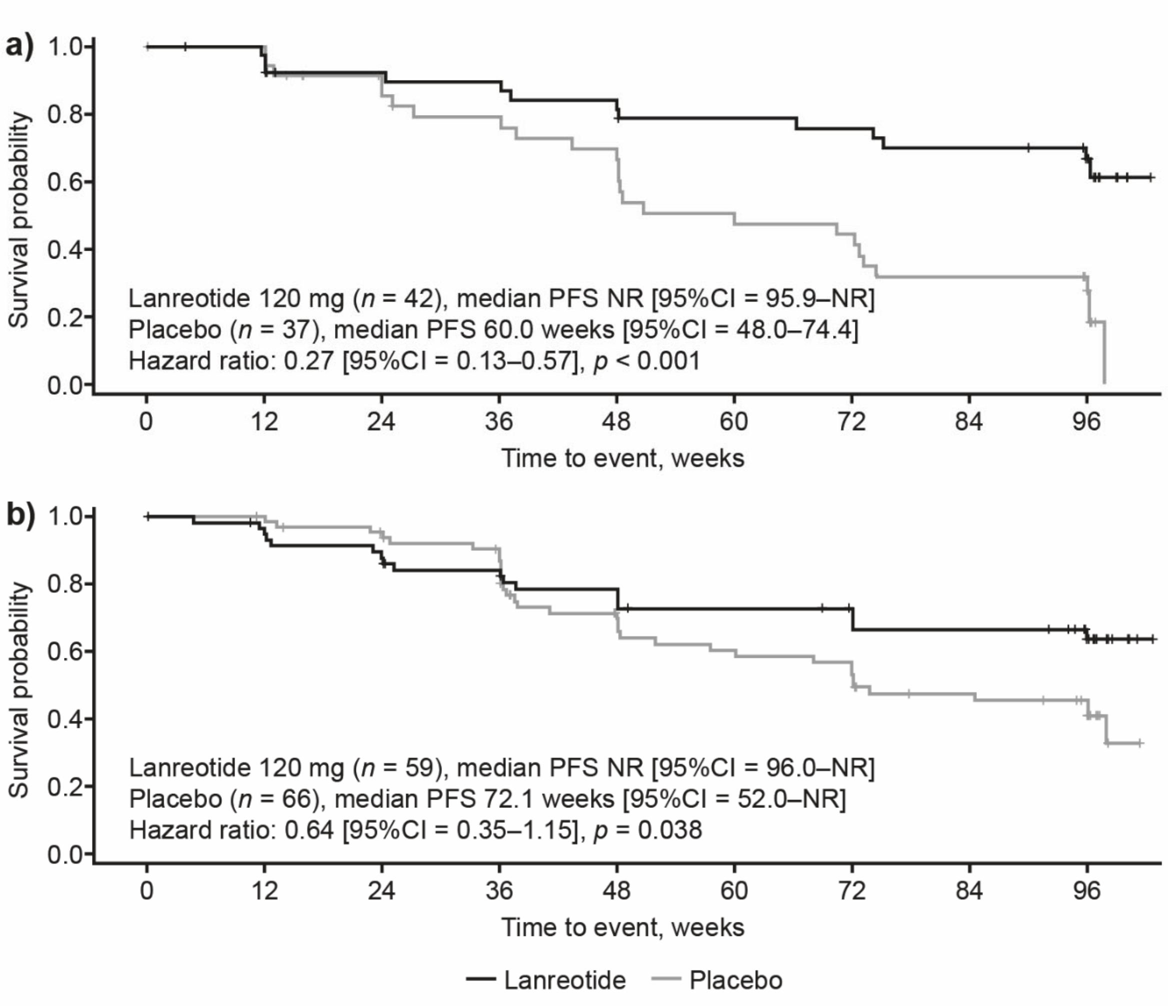

Figure S1. PFS by treatment group in patients (**A**) with DM and (**B**) without DM. CI, confidence interval; DM, diabetes mellitus; HR, hazard ratio; NR, not reached; PFS, progression-free survival.

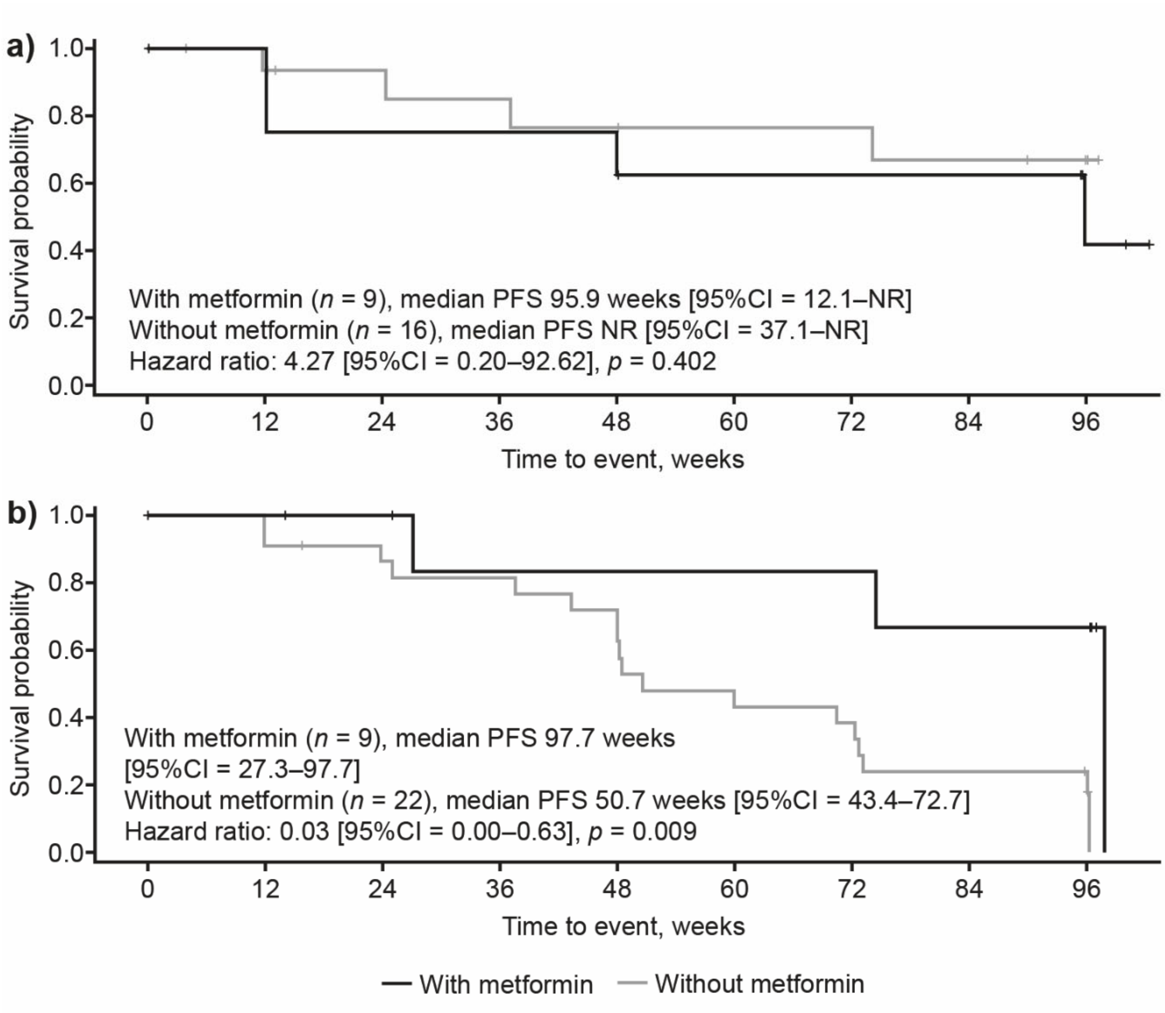

Figure S2. PFS by metformin treatment in all patients with DM who developed DM prior to study treatment and who received A) lanreotide 120 mg and B) placebo. CI, confidence interval; DM, diabetes mellitus; HR, hazard ratio; NR, not reached; PFS, progression-free survival. See Figure 3 for the landmark analysis (patients without disease progression at 3 months after treatment initiation) of these data.

Table S1. Baseline characteristics of patients with DM by metformin use (intention-to-treat population).

Characteristic	Lanreotide with metformin (*N* = 14)	Placebo with metformin (*N* = 10)	Lanreotide without metformin (*N* = 28)	Placebo without metformin (*N* = 27)
Sex, *n* (%)	*n* = 14	*n* = 10	*n* = 42	*n* = 27
Male	10 (71.4)	8 (80.0)	15 (53.6)	19 (70.4)
Female	4 (28.6)	2 (20.0)	13 (46.4)	8 (29.6)
Mean age (SD), in years	*n* = 14<br>62.8 (7.8)	*n* = 10<br>64.0 (8.2)	*n* = 28<br>63.0 (9.9)	*n* = 27<br>63.6 (8.3)
BMI, kg/m^2 (SD)	*n* = 12<br>30.6 (4.4)	*n* = 10<br>30.1 (4.6)	*n* = 27<br>29.3 (5.5)	*n* = 26<br>27.2 (6.5)
WHO performance status, *n* (%)	*n* = 14	*n* = 10	*n* = 28	*n* = 27
0 (normal activity)	10 (71.4)	8 (80.0)	26 (92.9)	22 (81.5)
1 (restricted activity)	4 (28.6)	1 (10.0)	2 (7.1)	5 (18.5)
2 (in bed <50% of the time)	0 (0.0)	1 (10.0)	0 (0.0)	0 (0.0)
Tumor grade, *n* (%)[a]	*n* = 14	*n* = 10	*n* = 28	*n* = 27
Grade 1	11 (78.6)	6 (60.0)	14 (50.0)	18 (66.7)

Grade 2	3 (21.4)	4 (40.0)	14 (50.0)	9 (33.3)
Primary tumor location, *n* (%)	*n* = 14	*n* = 10	*n* = 28	*n* = 27
Pancreas	9 (64.3)	5 (50.0)	15 (53.6)	20 (74.1)
Midgut	3 (21.4)	2 (20.0)	5 (17.9)	6 (22.2)
Hindgut	0	1 (10.0)	4 (14.3)	0 (0.0)
Other/unknown	2 (14.3)	2 (20.0)	4 (14.3)	1 (3.7)
Previous therapy for non-functioning enteropancreatic NETs at study entry, *n* (%)	*n* = 14<br>5 (35.7)	*n* = 10<br>1 (10.0)	*n* = 28<br>6 (21.4)	*n* = 27<br>2 (7.4)
Previous surgery of primary tumor, *n* (%)	*n* = 14<br>3 (21.4)	*n* = 10<br>3 (30.0)	*n* = 28<br>14 (50.0)	*n* = 27<br>9 (33.3)
Metformin daily dose (mg/day), mean (SD) [min, max]	*n* = 14<br>1371.4 (837.3)<br>[500.0; 3400.0]	*n* = 9<br>1690.7 (744.0)<br>[750.0; 3000.0]	–	–

BMI, body mass index; DM, diabetes mellitus; HbA_{1c}, glycated hemoglobin; HPF, high power fields; Ki67, proliferation index; NETs, neuroendocrine tumors; SD, standard deviation; WHO, World Health Organization. [a] Grade 1 = mitotic count <2 mitoses/10 HPF and/or Ki67 ≤2%; Grade 2 = mitotic count 2 to 20 mitoses/10 HPF and/or Ki67 >2% to 10%.

Table S2. List of Ethics Committees and/or Institutional Review Boards.

Country/ Sitenumber	Ethics Committee	Final Protocol, Version 22 Nov2005	Amended Protocol Incorporating Amendment 1 Version 03 Jan2007	Amended Protocol Incorporating Amendment 2 Version 30July 2007	Amended Protocol Incorporating Amendment 3 Version 30Apr2008	Amended Protocol Incorporating Amendment 4 Version 04Apr 2009	Amended Protocol Incorporating Amendment 5 Version 05Mar 2010	Amended Protocol Incorporating Amendment 6 Version 11Feb 2011	Amended Protocol Incorporating Amendment 7 Version 28Feb2012
Austria	Ethik-Kommission des Allgemeinen Krankenhauses und der Medizinischen Universität Wien Borschkegasse 8b/E06 1090 Wien	Date of Approval: NA	Date of Approval: 24 May 2007 Committee Chairperson Prof. Dr. E. Singer	Date of Approval: 06 Nov 2007 Committee Chairperson Prof. Dr. E. Singer	Date of Approval: 05 August 2008 Committee Chairperson Prof. Dr. E. Singer	Date of Approval: 28 July 2009 Committee Chairperson Prof. Dr. E. Singer	Date of Approval: 18 May 2010 Committee Chairperson Prof. Dr. E. Singer	Date of Approval: 10 May 2011 Committee Chairperson Prof. Dr. E. Singer	Date of Approval: 15 May 2012 Committee Vice Chairperson Prof. Dr. H. Greinix
Belgium 056001	Ethisch Comité Universitair Ziekenhuis De Pintelaan 185 9000 Gent Belgium	Date of Approval: NA (only Central EC approval in Belgium) Committee Chairperson: Prof. Dr. R. Rubens	Date of Approval: NA (only Central EC approval in Belgium) Committee Chairperson: Prof. Dr. R. Rubens	Date of Approval: NA (only Central EC approval in Belgium) Committee Chairperson: Prof. Dr. R. Rubens	Date of Approval: NA (only Central EC approval in Belgium) Committee Chairperson: Prof. Dr. R. Rubens	NA	NA	NA	NA
Belgium 056002	Commission d'éthique biomédicale Hospitalo-Facultaire Avenue Hippocrate 55.14 Tour Harvey - Niveau 0 1200 Bruxelles Belgium	Date of Approval: 04Sep2006 Committee Chairperson: Prof. Dr. J.M. Maloteaux	Date of Approval: 12Feb2007 Committee Chairperson: Prof. Dr. J.M. Maloteaux	Date of Approval: 07Sep2007 Committee Chairperson: Prof. Dr. J.M. Maloteaux	Date of Approval: 11Sep2008 Committee Chairperson: Prof. Dr. J.M. Maloteaux	Date of Approval: 09Sep2009 Committee Chairperson: Prof. Dr. J.M. Maloteaux	Date of Approval: 29Dec2010 Committee Chairperson: Prof. Dr. J.M. Maloteaux	Date of Approval: 31May2011 Committee Chairperson: Prof. Dr. J.M. Maloteaux	Date of Approval: 21May2012 Committee Chairperson: Prof. Dr. J.M. Maloteaux

Belgium 056003	Ethisch comité UZA Wilrijkstraat 10 2650 Edegem Belgium	Date of Approval: NA (only Central EC approval in Belgium) Committee Chairperson: Prof. Dr. P. Cras	Date of Approval: NA (only Central EC approval in Belgium) Committee Chairperson: Prof. Dr. P. Cras	Date of Approval: NA (only Central EC approval in Belgium) Committee Chairperson: Prof. Dr. P. Cras	Date of Approval: NA (only Central EC approval in Belgium) Committee Chairperson: Prof. Dr. P. Cras	Date of Approval: NA (only Central EC approval in Belgium) Committee Chairperson: Prof. Dr. P. Cras	Date of Approval: NA (only Central EC approval in Belgium) Committee Chairperson: Prof. Dr. P. Cras	Date of Approval: NA (only Central EC approval in Belgium) Committee Chairperson: Prof. Dr. P. Cras	Date of Approval: NA (only Central EC approval in Belgium) Committee Chairperson: Prof. Dr. P. Cras
Czech Rep 203001	Etická komise Vseobecne fakultni nemocnice v Praze Na Bojisti 1, 12808 Praha	Date of Approval: 16 Nov 2006 Committee Chairperson: MUDr. Josef Sedivy	Date of Approval: 15 Mar 2007 Committee Chairperson: MUDr. Josef Sedivy	Date of Approval: 20 Sep 2007 Committee Chairperson: MUDr. Josef Sedivy	Date of Approval: 18 Sep 2008 Committee Chairperson: MUDr. Josef Sedivy	Date of Approval: 16 Jul 2009 Committee Chairperson: MUDr. Josef Sedivy	Date of Approval: 02 Jun 2010 Committee Chairperson: MUDr. Josef Sedivy	Date of Approval: 23 Sep 2011 Committee Chairperson: MUDr. Josef Sedivy	Date of Approval: 21 Jun 2012 Committee Chairperson: MUDr. Josef Sedivy
Czech Rep 203002	Eticka komise Fakultni nemocnice Na Bulovce Budinova 2<br>180 00 Praha	N/A Submitted as part of Amendment 2	N/A Submitted as part of Amendment 2	Date of Approval: 09 Jan 2008 Committee Chairperson: Doc MUDr. Michal Holub	Date of Approval: 13 Aug 2008 Committee Chairperson: Doc MUDr. Michal Holub	Date of Approval: 08 Jul 2009 Committee Chairperson: Doc MUDr. Michal Holub	Date of Approval: 12 May 2010 Committee Chairperson: Doc MUDr. Michal Holub	Date of Approval: 11 Oct 2011 Committee Chairperson: Prof MUDr. Michal Holub	Date of Approval: 12 Jun 2012 Committee Chairperson: Prof MUDr. Michal Holub
Czech Rep 203003	Etická komise FN a LF UP Olomouc<br>I. P. Pavlova 6<br>775 20 Olomouc	N/A Submitted as part of Amendment 2	N/A Submitted as part of Amendment 2	Date of Approval: 10 Dec 2007 Committee Chairperson: Doc MUDr. Vladko Horcicka	Date of Approval: 15 Sep 2008 Committee Chairperson: Doc MUDr. Vladko Horcicka	Date of Approval: 13 Jul 2009 Committee Chairperson: Doc MUDr. Vladko Horcicka	Date of Approval: 10 May 2010 Committee Chairperson: Doc MUDr. Vladko Horcicka	N/A	N/A
Denmark	Regional Ethics Review Board in Region Midtjylland, Skottenborg 26, 8800 Viborg	Date of approval: 01 Jun 2007 Chairman Marie Bartholdy	Date of approval: 28 Jun 2007 Chairman Marie Bartholdy	Date of approval: 27 Nov 2007 Chairman Marie Bartholdy	Submitted in Denmark? No information found.	Submitted in Denmark? No information found.	Not submitted. Declaration of End of Trial submitted 16 Mar 2010	Not submitted. Declaration of End of Trial submitted 16 Mar 2010	Not submitted. Declaration of End of Trial submitted 16 Mar 2010

France 250	C.P.P. ILE DE FRANCE XI 20, rue Armaglis 78105 SAINT-GERMAIN EN LAYE CEDEX	Date of Approval: 23 Dec 2005 Committee Chairperson: Thierry De Rochegonde	Date of Approval: 29 Jan 2007 Committee Chairperson: Thierry De Rochegonde	Date of Approval: 27 Sep 2007 Committee Chairperson: Thierry De Rochegonde	Date of Approval: 12 Sep 2008 Committee Chairperson: Thierry De Rochegonde	Date of Approval: 19 June 2009 Committee Chairperson: Thierry De Rochegonde	Date of Approval: 05 July 2010 Committee Chairperson: Thierry De Rochegonde	Date of Approval: 08 July 2011 Committee Chairperson: Thierry De Rochegonde	Date of Approval: 16 Apr 2012 Committee Chairperson: Thierry De Rochegonde
Germany	Landesamt für Gesundheit und Soziales Ethik-Kommission des Landes Berlin Fehrbelliner Platz 1 10707 Berlin	Date of Approval: 05 April 2007 Committee Chairperson Prof. Dr. Edeltraud Garbe	Date of Approval: 25 May 2007 Committee Chairperson Prof. Dr. Edeltraud Garbe	Date of Approval: 24 October 2007 Committee Chairperson Prof. Dr. Edeltraud Garbe	Date of Approval: 12 August 2008 Committee Chairperson Prof. Dr. Edeltraud Garbe	Date of Approval: 03 July 2009 Committee Chairperson Prof. Dr. Edeltraud Garbe	Date of Approval: 07 May2010 Committee Chairperson Prof. Dr. Edeltraud Garbe	Date of Approval: 04 May 2011 Committee Chairperson Prof. Dr. Edeltraud Garbe	Date of Approval: 26 April 2012 Committee Chairperson Prof. Dr. Edeltraud Garbe
Greece 300001	National Ethics Committee, Mesogion 284, Chol-argos, 155 62	Date of Approval: 27 Mar 2007 Committee Chairperson: Prof D. Athanasiadis	N/A	N/A	N/A	N/A	N/A	N/A	N/A
India 356001	Human Ethics Committee, Tata Memorial Centre, Dr. E. Borges Marg, Parel, Mumbai 400012, Maharashtra, INDIA	N/A Submitted as part of Amendment 4	N/A Submitted as part of Amendment 4	N/A Submitted as part of Amendment 4	N/A Submitted as part of Amendment 4	Date of Approval: 20 Jan 2010 Committee Chairperson: Ms S. Barucha	Date of Approval: 28 Jun 2010 Committee Chairperson: Dr R C Mistry	Date of Approval: 03 Jan 2012 Committee Chairperson: Dr Tapan Saikla	Date of Approval: 30 Oct 2012 Committee Chairperson: Dr Tapan Saikla
India 356002	Institutional Ethics Committee, Global Hospitals 6-1-1070/1 to 4 Lakidi ka pool Hyderabad 500004 State: Andhra Pradesh India	N/A Submitted as part of Amendment 5	N/A Submitted as part of Amendment 5	N/A Submitted as part of Amendment 5	N/A Submitted as part of Amendment 5	N/A Submitted as part of Amendment 5	Date of Approval: 20 Oct 2010 Committee Chairperson: Justice Eshwar Prasad	Date of Approval: 19 Aug 2011 Committee Chairperson: Justice Eshwar Prasad	Date of Approval: 31 Jul 2012 Committee Chairperson: Justice Eshwar Prasad
Italy 380001	Fondazione IRCCS, Istituto Nazionale per lo	Date of Approval: 13 Sep 2006	Date of Approval: 08 Mar 2007	Date of Approval: 26 Sep 2007	Date of Approval: 13 Oct 2008	Date of Approval: 15 Jul 2009	Date of Approval: 20 Jul 2010	Date of Approval: 10 Jun 2011	Date of Approval: 05 Oct 2012

	Studio e la Cura dei Tumori, Via G Venezian 1, Milano 20133	Committee Chairperson: Dr Roberto Satolli	Committee Chairperson: Dr Roberto Satolli	Committee Chairperson: Dr Roberto Satolli	Committee Chairperson: Dr Roberto Satolli	Committee Chairperson: Dr Roberto Satolli	Committee Chairperson: Dr Roberto Satolli	Committee Chairperson: Dr Roberto Satolli	Committee Chairperson: Dr Roberto Satolli
Italy 380002	Azienda Sanitaria Ospedaliera 'San Giovanni Battista de Torino' C.so Bramanie 88/90 Torino 10126	Date of Approval: 19 Apr 2007 Committee Chairperson: Prof A. Pileri	Date of Approval: 30 Jul 2007 Committee Chairperson: Prof A. Pileri	Date of Approval: 25 Jul 2008 Committee Chairperson: Prof A. Pileri	Date of Approval: 14 Jan 2009 Committee Chairperson: Chairperson: Prof A. Pileri	Date of Approval: 29 Sep 2009 Committee Chairperson: Prof A. Pileri	Date of Approval: 19 Jul 2010 Committee Chairperson: Prof A. Pileri	Date of Approval: 07 Oct 2011 Committee Chairperson: Prof A. Pileri	Date of Approval: 21 Jan 2013 Committee Chairperson: Prof A. Pileri
Italy 380003	Centro di Riferimento Oncologico, Istituto Nazionale dei Tumori, Via Pedemontana Occidentale 12, Aviano 33081	Date of Approval: 11 Dec 2006 Committee Chairperson: Luciano Padovese	Date of Approval: 09 March 2007 Committee Chairperson: Luciano Padovese	Date of Approval: 27 Nov 2007 Committee Chairperson: Luciano Padovese	Date of Approval: 30 Sep 2008 Committee Chairperson: Luciano Padovese	Date of Approval: 05 Aug 2009 Committee Chairperson: Luciano Padovese	Date of Approval: 02 Sep 2010 Committee Chairperson: Luciano Padovese	Date of Approval: 20 May 2011 Committee Chairperson: Luciano Padovese	Date of Approval: 04 Jul 2012 Committee Chairperson: Luciano Padovese
Italy 380004	A.O.U.P. S. Orsola- Malpighi di Bologna, Via Albertoni 15, Bologna 40138	Date of Approval: 07 Nov 2006 Committee Chairperson: Dr Maridelma Canova	Date of Approval: 24 Apr 2007 Committee Chairperson: Prof Fabrizio de Ponti	Date of Approval: 22 Nov 2007 Committee Chairperson: Prof Fabrizio de Ponti	Date of Approval: 10 Dec 2008 Committee Chairperson: Prof Fabrizio de Ponti	Date of Approval: 06 Oct 2009 Committee Chairperson: Prof Fabrizio de Ponti	Date of Approval: 22 Sep 2010 Committee Chairperson: Prof Nicola Montanaro	Date of Approval: 20 Jun 2011 Committee Chairperson: Prof Nicola Montanaro	Date of Approval: 17 Jul 2012 Committee Chairperson: Prof Nicola Montanaro
Italy 380005	A.O.U. Pisana Via Roma 67, Pisa 56126	Date of Approval: 14 Dec 2006 Committee Chairperson: Prof Roberto Barsotti	Date of Approval: 01 Jul 2007 Committee Chairperson: Prof Romano Danesi	Date of Approval: 31 Jan 2008 Committee Chairperson: Prof Romano Danesi	NA closed site	NA closed site	NA closed site	NA closed site	NA closed site
Italy 380007	Centro di Riferimento Oncologico, Istituto Nazionale dei Tumori, Via Pedemontana Occidentale 12, Aviano 33081	Date of Approval: 11 Dec 2006 Committee Chairperson:	Date of Approval: 09 March 2007 Committee Chairperson:	Date of Approval: 27 Nov 2007 Committee Chairperson:	Date of Approval: 30 Sep 2008 Committee Chairperson:	Date of Approval: 05 Aug 2009 Committee Chairperson:	Date of Approval: 02 Sep 2010 Committee Chairperson:	Date of Approval: 20 May 2011 Committee Chairperson:	Date of Approval: 04 Jul 2012 Committee Chairperson:

		Luciano Padovese	Luciano Padovese	Luciano Padovese	Luciano Padovese	Luciano Padovese	Luciano Padovese	Luciano Padovese	Luciano Padovese
Italy 380008	Universita degli Studi Ferderico II di Napoli, via Pansini 5, Napoli 80131	Date of Approval: 12 Mar 2007 Committee Chairperson: Prof. Claudio Buccelli	Date of Approval: 13 Jul 2007 Committee Chairperson: Prof. Claudio Buccelli	Date of Approval: 15 Feb 2008 Committee Chairperson: Prof. Claudio Buccelli	Date of Approval: 16 Dec 2008 Committee Chairperson: Prof. Claudio Buccelli	Date of Approval: 16 Jul 2009 Committee Chairperson: Prof. Claudio Buccelli	Date of Approval: 23 Mar 2011 Committee Chairperson: Prof. Claudio Buccelli	Date of Approval: 01 Jun 2011 Committee Chairperson: Prof. Claudio Buccelli	Date of Approval: 25 Jan 2013 Committee Chairperson: Prof. Claudio Buccelli
Netherlands 528001	Medisch Ethische Toetsings Commissie Erasmus MC DrMolewaterplein40 3015 GD Rotterdam Netherlands	Date of Approval: 21 November 2006 Committee Chairperson: Prof. Dr. H.W. Tilanus	Date of Approval: 03 May 2007 Committee Chairperson: Prof. Dr. H.W. Tilanus	Date of Approval: 03 October2007 Committee Chairperson: Prof. Dr. H.W. Tilanus	N/A	N/A	N/A	N/A	N/A
Netherlands 528002	Medisch Ethische Toetsings Commissie Universitair Medisch Centrum Groningen De Brug kamer 07.067 9700 RB Groningen Netherlands	Date of Approval: NA (only Central EC approval in Netherlands) Committee Chairperson: NA	Date of Approval: NA (only Central EC approval in Netherlands) Committee Chairperson: NA	Date of Approval: NA (only Central EC approval in Netherlands) Committee Chairperson: NA	N/A	N/A	N/A	N/A	N/A
Netherlands 528003	Medisch Ethische Toetsings Commissie Universitair Medisch Centrum Utrecht Heidelbergglaan 100 Postbus 85500 3508 GA Utrecht Netherlands	Date of Approval: NA (only Central EC approval in Netherlands) Committee Chairperson: NA	Date of Approval: NA (only Central EC approval in Netherlands) Committee Chairperson: NA	Date of Approval: NA (only Central EC approval in Netherlands) Committee Chairperson: NA	N/A	N/A	N/A	N/A	N/A
Poland CEC	Slaskiego Uniwersytetu Medycznego w Katowicach, ul. Poniatowskiego 15, Katowice 40-055	N/A – site not open	Date of Approval: 07 Feb 2007 Committee Chairperson:	Date of Approval: 08 Nov 2007 Committee Chairperson:	Date of Approval: 29 Sep 2008 Committee Chairperson:	Date of Approval: 17 Nov 2009 Committee Chairperson:	Date of Approval: 06 Jul 2010 Committee Chairperson:	Date of Approval: 20 Sep 2011 Committee Chairperson:	Date of Approval: 05 Jun 2012 Committee Chairperson: Prof dr hab

			Prof Stefan Kossmann	Prof Stefan Kossmann	Prof Stefan Kossmann	Prof Stefan Kossmann	Prof Stefan Kossmann	Prof Stefan Kossmann	Maria Trusz-Gluza
Slovakia 703001	Vychodoslovensky Onkologicky ustav a.s., P.O. Box D-47, Rastislavova 43, Kosice 04191	N/A Submitted as part of Amendment 2	N/A Submitted as part of Amendment 2	Date of Approval: 19 Dec 2007	Date of Approval: 09 Sep 2008 Committee Vice Chairperson: Tibor Packan, MD	Date of Approval: 27 Jul 2009 Committee Vice Chairperson: Tibor Packan, MD	N/A	N/A	N/A
Slovakia 703002	Eticka komisia, Narodny onkologicky ustav, Klenova 1, 833 10 Bratislava, Slovakia	N/A Submitted as part of Amendment 2	N/A Submitted as part of Amendment 2	Date of Approval: 11 Dec 2007	Date of Approval: 10 Sep 2008 Committee Chairperson: Kristina Krizanova MD	Date of Approval: 08 Jul 2009 Committee Chairperson: Kristina Krizanova MD	Date of Approval: 12 May 2010 Committee Chairperson: Kristina Krizanova MD	Date of Approval: 15 Jun 2011 Committee Chairperson: Kristina Krizanova MD	Date of Approval: 17 May 2012 Committee Chairperson: Kristina Krizanova MD
Spain 724001	CEC: Comité Ético de Investigación Clínica of Hospital Universitari Vall d'Hebron Institut de Recerca 3ª Planta Passeig de la Vall d'Hebron 119-129 08035 Barcelona Spain	Date of Approval: 15 Sep 2006 Committee Chairperson: Dr. Gaieta Permanyer Miralda	Date of Approval: 13 Mar 2007 Committee Chairperson: Dr. Gaieta Permanyer Miralda	Date of Approval: 09 Oct 2007 Committee Chairperson: Dr. Francisco Latorre Arteche	Date of Approval: 10 Oct 2008 Committee Chairperson: Dr. Francisco Latorre Arteche	Date of Approval: 10 Jul 2009 Committee Chairperson: Dr. Francisco Latorre Arteche	Date of Approval: 04 Jun 2010 Committee Chairperson: Dr. Francisco Latorre Arteche	Date of Approval: 10 Jun 2011 Committee Chairperson: Dr. Soledad Gallego Melcón	Date of Approval: 08 Jun 2012 Committee Chairperson: Dr. Soledad Gallego Melcón
Spain 724002	CEC of site 724001 and the following LEC: CEIC Hospital Universitario de Bellvitge, Edificio Unitat de Recerca, Feixa Llarga, s/n 08907 L'Hospitalet de Llobregat, Barcelona, Spain	Date of Approval: 15 Sep 2006 Committee Chairperson: Dr. Gaieta Permanyer Miralda	Date of Approval: 13 Mar 2007 Committee Chairperson: Dr. Gaieta Permanyer Miralda	Date of Approval: 09 Oct 2007 Committee Chairperson: Dr. Francisco Latorre Arteche	Date of Approval: 10 Oct 2008 Committee Chairperson: Dr. Francisco Latorre Arteche	Date of Approval: 10 Jul 2009 Committee Chairperson: Dr. Francisco Latorre Arteche	Date of Approval: 04 Jun 2010 Committee Chairperson: Dr. Francisco Latorre Arteche	Date of Approval: 10 Jun 2011 Committee Chairperson: Dr. Soledad Gallego Melcón	Date of Approval: 08 Jun 2012 Committee Chairperson: Dr. Soledad Gallego Melcón
Spain 724005	CEC of site 724001 and the following LEC: CEIC A1 Hospital General Universitario Gregorio Marañón	Date of Approval: 15 Sep 2006 Committee Chairperson:	Date of Approval: 13 Mar 2007 Committee Chairperson:	Date of Approval: 09 Oct 2007 Committee Chairperson: Dr. Francisco	Date of Approval: 10 Oct 2008 Committee Chairperson: Dr. Francisco	Date of Approval: 10 Jul 2009 Committee Chairperson: Dr. Francisco	Date of Approval: 04 Jun 2010 Committee Chairperson: Dr. Francisco	Date of Approval: 10 Jun 2011 Committee Chairperson:	Date of Approval: 08 Jun 2012 Committee Chairperson: Dr. Soledad

	Pabellón de Gobierno, Dr. Esquerdo 46 28007 Madrid, Spain	Dr. Gaieta Perrmanyer Miralda	Dr. Gaieta Perrmanyer Miralda	Latorre Arteche	Latorre Arteche	Latorre Arteche	Latorre Arteche	Dr. Soledad Gallego Melcón	Gallego Melcón
Spain 724006	CEC of site 724001 and the following LEC: CEIC Hospital Universitario La Paz Paseo de la Castellana, 261 Escuela de Enfermería Planta 4ª Despacho 424 28046 Madrid, Spain	Date of Approval: 15 Sep 2006 Committee Chairperson: Dr. Gaieta Perrmanyer Miralda	Date of Approval: 13 Mar 2007 Committee Chairperson: Dr. Gaieta Perrmanyer Miralda	Date of Approval: 09 Oct 2007 Committee Chairperson: Dr. Francisco Latorre Arteche	Date of Approval: 10 Oct 2008 Committee Chairperson: Dr. Francisco Latorre Arteche	Date of Approval: 10 Jul 2009 Committee Chairperson: Dr. Francisco Latorre Arteche	Date of Approval: 04 Jun 2010 Committee Chairperson: Dr. Francisco Latorre Arteche	Date of Approval: 10 Jun 2011 Committee Chairperson: Dr. Soledad Gallego Melcón	Date of Approval: 08 Jun 2012 Committee Chairperson: Dr. Soledad Gallego Melcón
Spain 724007	CEC of site 724001 and the following LEC: Comité Ético de Investigación Clínica Hospital Universitario Nuestra Sra. De Candelaria Unidad de Calidad (3ª planta de la Residencia General, nº 331) Carretera del Rosario, 145 38010 Santa Cruz de Tenerife, Spain	Date of Approval: 15 Sep 2006 Committee Chairperson: Dr. Gaieta Perrmanyer Miralda	Date of Approval: 13 Mar 2007 Committee Chairperson: Dr. Gaieta Perrmanyer Miralda	Date of Approval: 09 Oct 2007 Committee Chairperson: Dr. Francisco Latorre Arteche	Date of Approval: 10 Oct 2008 Committee Chairperson: Dr. Francisco Latorre Arteche	Date of Approval: 10 Jul 2009 Committee Chairperson: Dr. Francisco Latorre Arteche	Date of Approval: 04 Jun 2010 Committee Chairperson: Dr. Francisco Latorre Arteche	Date of Approval: 10 Jun 2011 Committee Chairperson: Dr. Soledad Gallego Melcón	Date of Approval: 08 Jun 2012 Committee Chairperson: Dr. Soledad Gallego Melcón
Sweden	Regional Ethical Review Board in Uppsala, Drottninggatan 4, 753 09 Uppsala	Date of approval: 04 Apr 2007 Chairman Erik Lempert	Date of approval: 28 Jun 2007 Chairman Erik Lempert	Date of approval: 20 Nov 2007 Chairman Erik Lempert	Date of approval: 06 May 2009 Chairman Erik Lempert	Date of approval: 02 Jul 2009 Chairman Erik Lempert	Date of approval: 19 May 2010 Chairman Erik Lempert	Date of approval: 16 Jun 2011 Chairman Erik Lempert	Submitted for information only since there were no active patients in Sweden. AoR 14 Apr 2012.
UK	NRES Committee London-South East, South East Coast Strategic Health Authority, Preston Hall, Aylesford, Kent ME20 7NJ	Date of Approval: 14 Aug 2006 Committee Chairperson: Dr J M Lamberty	Date of Approval: 01 Feb 2007 Committee Chairperson: Dr L A Ruben	Date of Approval: 23 Aug 2007 Committee Chairperson: Dr J M Lamberty	Date of Approval: 25 Jul 2008 Committee Chairperson: Dr L A Ruben	Date of Approval: 26 Jun 2009 Committee Chairperson: Dr L A Ruben	Date of Approval: 05 May 2010 Committee Chairperson: Dr L A Ruben	Date of Approval: 26 May 2011 Committee Chairperson: Dr L A Ruben	N/A
USA 840001	Providence Portland Medical Center	N/A	N/A	Date of Approval:	Date of Approval:	Date of Approval:	N/A	N/A	N/A

	Institutional Review Board<br>5251 NE Glisan St., Bldg A, 3rd Floor<br>Portland, OR 97213			18 June 2008<br>Committee Chairperson: Eric Friedman, M.D.	26 August 2008<br>Committee Chairperson: Eric Friedman, M.D.	23 June 2009<br>Committee Chairperson: Eric Friedman, M.D.			
USA 840002	Western Institutional Review Board (WIRB)<br>3535 Seventh Ave. SW<br>PO Box 12029<br>Olympia, WA 98502-20209	N/A	N/A	Date of Approval: 18 August 2008<br>Committee Chairperson: Theodore D. Schultz, M.D.	Date of Approval:<br>07 October<br>2008<br>Committee Chairperson: Theodore D. Schultz, M.D.	N/A	N/A	N/A	N/A
USA 840003	The University of Texas M.D. Anderson Cancer Center Institutional Review Board<br>1515 Holcombe Blvd., Unit 1437<br>Houston, TX 77030	N/A	N/A	N/A	Date of Approval: 25 September 2008<br>Committee Chairperson: *IRB does not disclose member names	Date of Approval: 22 May 2009<br>Committee Chairperson: *IRB does not disclose member names	Date of Approval: 25 May 2010<br>Committee Chairperson: *IRB does not disclose member names	Date of Approval: 12 May 2011<br>Committee Chairperson: *IRB does not disclose member names	Date of Approval: 10 September 2012<br>Committee Chairperson: *IRB does not disclose member names
USA 840004	Johns Hopkins Medicine<br>IRB<br>Reed Hall B-130<br>1620 McElderry St. Baltimore, MD 21205- 1911	N/A	N/A	Date of Approval: 30 October 2008<br>Committee Chairperson: David Cornblath, M.D.	Date of Approval: 08 January 2009<br>Committee Chairperson: David Cornblath, M.D.	Date of Approval:<br>18 May 2009<br>Committee Chairperson: David Cornblath, M.D.	Date of Approval:<br>29 April 2010<br>Committee Chairperson: David Cornblath, M.D.	Date of Approval:<br>7 July 2011<br>Committee Chairperson: David Cornblath, M.D.	Date of Approval: 06 September 2012<br>Committee Chairperson: Kenneth Cohen, M.D.
USA 840005	Cedars-Sinai Medical Center	N/A	N/A	Date of Approval:	Date of Approval:	Date of Approval:<br>05 June 2009	Date of Approval:<br>04 May 2010	Date of Approval:<br>24 May 2011	Date of Approval: 26 September 2012

	Office of Research Compliance<br>8383 Wilshire Blvd., Suite 742<br>Beverly Hills, CA 90211			23 October 2008<br>Committee Chairperson:	06 January 2009<br>Committee Chairperson: Ilana Cass, M.D.	Committee Chairperson: Ilana Cass, M.D.	Committee Chairperson: Scott Cunneen, M.D.	Committee Chairperson: Scott Cunneen, M.D.	Committee Chairperson: Scott Cunneen, M.D.
USA 840007	Roger Wilson, MD<br>Chairman<br>Institutional Review Board<br>Memorial Sloan-Kettering Cancer Center<br>1275 York Avenue New York, NY 10065	N/A	N/A	N/A	Date of Approval:<br>30 October 2008<br>Committee Chairperson: Roger S. Wilson, M.D.	Date of Approval:<br>27 May 2009<br>Committee Chairperson: Roger S. Wilson, M.D.	N/A	N/A	N/A
USA 840009	Institutional Review Board<br>Dana-Farber Cancer Institute<br>Dana-Farber/Harvard Cancer Center<br>44 Binney Street<br>OS229<br>Boston, MA 02115	N/A	N/A	N/A	N/A	N/A	Date of Approval:<br>29 March 2011<br><br>Committee Chairperson:<br>*IRB does not release names of members	N/A	N/A
USA 840010	OHSU Regulatory Integrity Office (ORIO)<br>3181 SW Sam Jackson Park Road<br>Portland, OR 97239	N/A	N/A	N/A	N/A	N/A	N/A	N/A	N/A
